# Does the Duration of Primary and First Revision Surgery Influence the Probability of First and Subsequent Implant Failures after Extremity Sarcoma Resection and Megaprosthetic Reconstruction?

**DOI:** 10.3390/cancers13112510

**Published:** 2021-05-21

**Authors:** Christoph Theil, Kristian Nikolaus Schneider, Georg Gosheger, Ralf Dieckmann, Niklas Deventer, Jendrik Hardes, Tom Schmidt-Braekling, Dimosthenis Andreou

**Affiliations:** 1 Department of General Orthopedics and Tumor Orthopedics, Muenster University Hospital, Albert-Schweitzer Campus 1, 48149 Muenster, Germany; kristian.schneider@ukmuenster.de (K.N.S.); georg.gosheger@ukmuenster.de (G.G.); r.dieckmann@bk-trier.de (R.D.); niklas.deventer@ukmuenster.de (N.D.); Jendrik.hardes@uk-essen.de (J.H.); tom.schmidt-braekling@ukmuenster.de (T.S.-B.); Dimosthenis.andreou@helios-gesundheit.de (D.A.); 2 Department of Orthopedics, Krankenhaus der Barmherzigen Brueder, Nordallee 1, 54292 Trier, Germany; 3 Department of Musculoskeletal Oncology, Essen University Hospital, Hufelandstraße 55, 45147 Essen, Germany; 4 Department of Orthopedic Oncology and Sarcoma Surgery, Sarcoma Centre Berlin-Brandenburg, Helios Klinikum Bad Saarow, 15526 Bad Saarow, Germany

**Keywords:** tumor endoprosthesis, megaprosthesis, periprosthetic joint infection, revision surgery, sarcoma

## Abstract

**Simple Summary:**

Tumor endoprostheses are a common type of reconstruction after the resection of an extremity bone sarcoma. However, in the long-term, first and subsequent implant failures leading to revision surgery are common. One potential risk factor for implant failure is the length of surgery. This study investigates the impact of the length of surgery on prosthetic survival in 568 patients with sarcoma. Patients who had a first implant failure had a longer surgery; however, there were no differences in the infection-free survival, but only in the probability of mechanical failure. Patients with a subsequent revision surgery for infection had a shorter duration of surgery during the first revision. In conclusion, a shorter surgery appears beneficial; however, longer surgeries are not clearly associated with infection. In revision surgery, a longer operating time, indicating a more thorough debridement, may be desirable.

**Abstract:**

Complications in megaprosthetic reconstruction following sarcoma resection are quite common. While several risk factors for failure have been explored, there is a scarcity of studies investigating the effect of the duration of surgery. We performed a retrospective study of 568 sarcoma patients that underwent megaprosthetic reconstruction between 1993 and 2015. Differences in the length of surgery and implant survival were assessed with the Kaplan–Meier method, the log-rank test and multivariate Cox regressions using an optimal cut-off value determined by receiver operating curves analysis using Youden’s index. 230 patients developed a first and 112 patients a subsequent prosthetic failure. The median duration of initial surgery was 210 min. Patients who developed a first failure had a longer duration of the initial surgery (225 vs. 205 min, *p* = 0.0001). There were no differences in the probability of infection between patients with longer and shorter duration of initial surgery (12% vs. 13% at 5 years, *p* = 0.492); however, the probability of mechanical failure was higher in patients with longer initial surgery (38% vs. 23% at 5 years, *p* = 0.006). The median length of revision surgery for the first megaprosthetic failure was 101 min. Patients who underwent first revision for infection and did not develop a second failure had a longer median duration of the first revision surgery (150 min vs. 120 min, *p* = 0.016). A shorter length of the initial surgery appears beneficial, however, the notion that longer operating time increases the risk of deep infection could not be reproduced in our study. In revision surgery for infection, a longer operating time, possibly indicating a more thorough debridement, appears to be associated with a lower risk for subsequent revision.

## 1. Introduction

The use of megaprostheses to address osteoarticular defects after limb-sparing resections of malignant bone tumors has become the reconstruction method of choice over the last few decades [1,2,3]. As the prognosis of extremity sarcoma patients has improved over the last few decades [1,4] more and more patients require revision surgeries for—sometimes multiple—implant failures [1,3,5]. These revisions are associated with a high disease burden [1,6] and may potentially result in the amputation of the affected limb [7,8]. As a result, there is a need to identify potential risk factors for the development of prosthetic failures that ideally would be accessible for perioperative optimization.

Previous studies on non-megaprosthetic arthroplasty have identified a longer duration of surgery as a potential risk factor for complications [9,10], possibly because the bacterial contamination of the surgical field might increase with the length of surgery [11]. Consequently, the impact of the length of surgery has also been discussed as a risk factor for megaprosthetic infection [12]. However, only very few studies have, to our knowledge, investigated this issue [13,14,15], with some studies finding an association between a longer surgical time and a higher probability for infections, while another did not. However, these studies were limited by the small number of implants included, while their findings were based on univariate analyses, despite the fact that several factors, such as reconstruction length may interact with the duration of a procedure. Furthermore, subsequent prosthetic failures and re-revision surgeries have become more common in oncological patients [1,3,16,17], but no study has yet investigated the impact of the length of the first revision surgery on the probability of subsequent failures.

We therefore asked whether there is an association between the length of primary or revision surgery at a tertiary bone sarcoma center and the probability of mechanical or infectious megaprosthetic complications. We hypothesized that a longer duration of a procedure might be a risk factor for further complications, especially infections.

## 2. Materials and Methods

### 2.1. Study Design

We retrospectively queried our institution’s database and identified 817 patients with bone tumors who underwent resection and megaprosthetic reconstruction of the upper or lower limb using a single modular system (MUTARS^TM^, Implantcast GmbH, Buxtehude, Germany) between 1993 and 2015. Patient demographics, tumor characteristics, surgical and oncological treatments, postoperative complications and their treatment as well as patient follow-up and oncological outcomes were retrospectively collected from the patients’ medical records and entered into an electronic datasheet. All patient data were anonymized before analysis.

**Figure 1:**
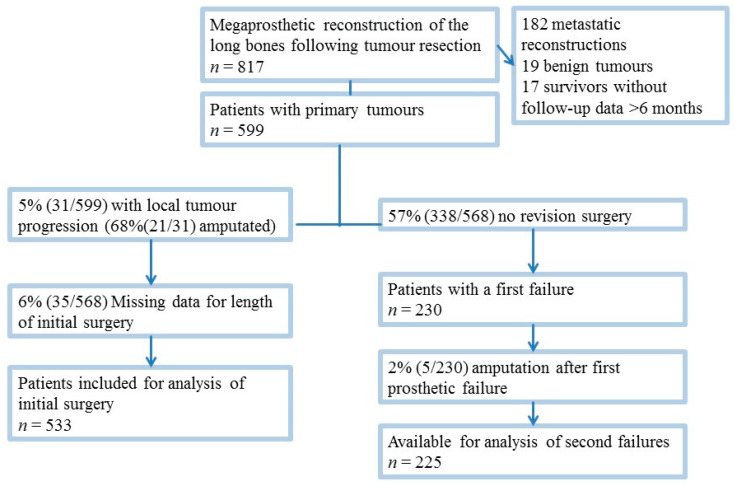
STROBE diagram showing inclusion of patients.

Patients with bone metastases, benign tumors as well as surviving patients with follow-up of less than 6 months, who were considered to be lost to follow-up, were excluded from this analysis (Figure 1). We also excluded patients undergoing revisions due to a tumor recurrence/progression, as they can potentially be associated with a multitude of factors that were not comprehensively investigated in the present study (e.g., tumor size, histology, neo-/adjuvant treatments, response etc.).

Furthermore, all patients who underwent amputation to treat a first prosthetic failure (2%, 5/230) were excluded from analysis of second failures.

The length of surgery was defined as the time from the first incision to wound closure and was retrieved from the operating theatre records. For patients with two-stage revisions the duration of the longest procedure was recorded. Patients with missing data regarding the length of the initial surgery or surgical treatment for the first complication were excluded from the respective analyses (Figure 1). As a result, 533 patients were available to evaluate whether the length of initial surgery was associated with the development of a first prosthetic failure and 225 patients were available to assess a possible association between the length of revision surgery and the development of a second failure (Figure 1). The median follow-up was 55 months (interquartile range (IQRI 25–114) for all patients and 68 months (IQR 35–127) for surviving patients. The median follow-up for patients who developed a first prosthetic failure amounted to 91 months (IQR 45–159) for all patients and 99 months (IQR 55–170) for surviving patients. Patient, tumor, and treatment characteristics of these patients are presented in Table 1 and Table 2.

First and subsequent failures were classified according to the system proposed by Henderson et al. [18]. For further analysis, infections were looked at in a subgroup analysis and compared to non-infectious failures. Infections were treated using debridement, antibiotics, irrigation and implant retention (DAIR), one-stage or two-stage exchanges depending on the type of infection (early or late), soft tissue condition, stem ingrowth, culture results as well as the quality and amount of residual bone.

### 2.2. First and Second Implant Failures

There was a total of 230 first implant failures after a median time of 17 months (IQR 4–60), among which structural failures were found in 15% (84/568) (Table S1), followed by infection in 10% of cases (58/568), aseptic loosening in 8% of cases (45/568) and soft tissue failures in 7% of cases (43/568). The overall revision-free implant survival probability was 74% (95% CI 70–78) after two years and 64% (95% CI 60–68) at five years. The infection-free survival probability was 91% (95% CI 88–94) after two years and 87% (95% CI 84–90) after five years, while the survivorship free from revision for a mechanical failure was 83% (95% CI 80–86) after two years and 73% (95% CI 69–77) after five years. Five of these patients underwent amputation to treat the first complication and were excluded from the analysis of second failures. Among the remaining 225 patients, 50% (112/225) had a second failure after a median time of 17 months (IQR 5–47) (20% infections (45/112), followed by 17% structural failures (38/112), 8% aseptic loosening (19/112), and 3% soft tissue failures (7/112). The implant survivorship free from revision for a second failure was 69% (95% CI 63–75%) after two years and 46% after five years (95% CI 38–53) following the first revision surgery.

### 2.3. Statistical Analysis

The duration of follow-up and time to implant failure were calculated from the date of the primary tumor surgery. The time to second failure was calculated from the date of final reconstruction for the previous failure. Contingency tables were analyzed using the chi-squared test. Continuous variables were checked for normality using the Shapiro–Wilk test. Medians with IQRs were calculated for non-parametric data. Non-parametric analyses were performed using the Mann–Whitney U-Test. Implant survival probabilities, with their respective 95% confidence intervals, were calculated with the Kaplan–Meier method and compared using the log-rank test. We used receiver operating characteristic (ROC) curves to analyze the association between the length of surgery and implant failure. Area under the curve (AUC) values were calculated using a non-parametric distribution assumption. The optimal cut-off value was determined using the Youden index. Hazard ratios (HR) were estimated with their respective 95% confidence intervals (CI) in multivariate Cox regression models. Multivariate analysis of risk factors was conducted including risk factors that were identified from univariate analysis and taking into consideration the findings of a previous study on subsequent failures [3].

Statistical calculations were performed with SPSS Version 25.0 (IBM Corp., Armonk, NY, USA). All *p* values were two-sided; a *p*-value < 0.05 was considered significant.

## 3. Results

### 3.1. First Implant Failures

The median length of tumor resection and megaprosthetic reconstruction was 210 min (IQR 174–255). Patients who developed an implant failure had a longer median duration of the initial surgery compared to patients with no failures (225 min (IQR 180–268) vs. 205 min (IQR 160–242), *p* = 0.0001). Contrary to our hypothesis, subgroup analysis showed that patients who developed an infection as a first failure did not have a longer primary surgery time compared to patients with no infections (210 min (IQR 173–255) vs. 200 min (IQR 170–248), *p* = 0.417). On the other hand, patients treated for a mechanical complication had a significantly longer primary surgery time compared to patients with no mechanical complications (235 min (IQR 185–278) vs. 204 (IQR 160–243), *p* = 0.0001). As there are relevant differences in median operating times for different anatomic locations, subgroup analyses were performed and presented in Table 3.

The ROC analysis showed a significant association between the length of primary surgery and first implant failure (AUC 0.592, 95% CI 0.543–0.641, *p* = 0.0001) with an optimal cut-off at 234 min. Survivorship free from revision was significantly higher in patients with a shorter surgical time (68% (95% CI 62–74) vs. 55% (95% CI 47–63) at five years, *p* = 0.036). Again, subgroup analyses showed no differences in the infection-free implant survival probability between patients with shorter and longer durations of primary surgery (88% (95% CI 83–93) vs. 87% (95% CI 83–92), *p* = 0.492). On the other hand, patients with shorter durations of primary surgery had a significantly higher implant survivorship free from revision for a mechanical failure compared to patients with longer surgical durations (77% (95% CI 72–83) vs. 62% (95% CI 54–70) at 5 years, *p* = 0.006). Multivariate analysis (Table 4) confirmed that the length of the initial surgical procedure was a significant risk factor for first implant failure taking potential further risk factors into consideration.

### 3.2. Second Implant Failure

The median duration of tumor resection and megaprosthetic reconstruction in patients who developed a first implant failure was 218 min (IQR 180–261). In this cohort, we found no differences in the length of the primary surgery between patients who developed a second implant failure and patients who did not (218 (IQR 180–255) vs. 220 (IQR 180–274), *p* = 0.261).

The median length of revision surgery for the first megaprosthetic failure was 101 min (IQR 64–153). Interestingly, patients who suffered a second failure had a shorter duration of revision surgery compared to patients who had no further failures (90 min (IQR 55–128) vs. 117 min (IQR 75–157), *p* = 0.014). Subgroup analyses in this cohort showed that there were no significant differences in the length of revision surgery between patients treated for a mechanical first complication (median 95 min (IQR 64–152) vs. 85 min (IQR 52–121), *p* = 0.184), whereas patients who underwent first revision for an infection and did not develop a second implant failure and had a significantly longer median duration of the first revision surgery (150 min (IQR 118–186) vs. 120 min (85–150), *p* = 0.016).

ROC analysis confirmed an association between a shorter length of revision surgery and a second implant failure (AUC 0.398, 95% CI 0.317–0.478), *p* = 0.014) with an optimal cut-off at 123 min. The implant survivorship free from revision for a second failure was significantly higher in patients with a longer duration of revision surgery (62% (95% CI 48–76) vs. 39% (95% CI 28–50) at 5 years after first revision surgery, *p* = 0.004).

In multivariate analysis (Table 5), a longer duration of the revision surgery was associated with a reduced risk for second complications.

## 4. Discussion

Patients who undergo extremity sarcoma resection and megaprosthetic reconstruction are at a high risk for prosthetic failure and subsequent revisions [3,18,19]. Orthopaedic oncologists are therefore required to evaluate possible risk factors for failure and, ideally, identify areas of optimization potential. The length of surgery as a potential risk factor has been studied previously [20]; however, previous results have been inconclusive [13,14,15]. As sarcoma resection and megaprosthetic reconstructions have a longer surgical duration and are associated with a higher risk of failure compared to non-oncological arthroplasty procedures [18,21], modifying a procedure related risk factor, such as the duration of the surgery, would offer surgeons a chance to reduce the burden of megaprosthetic revision. Our study investigated the influence of the length of the initial and first revision surgery on first and subsequent megaprosthetic failures in sarcoma patients. While we found that a longer operating time in the initial surgery was generally associated with shorter revision free survival probability, it was not associated, as we expected, with a higher infection risk, but with a higher probability of non-infectious failures. On the other hand, in patients who underwent revision surgery for periprosthetic infection, a shorter duration of the revision surgery was associated with a higher risk of subsequent failures.

The results of our study should be interpreted taking its limitations into consideration. Given its retrospective design, we extracted available data from patients’ records, resulting in a possible selection bias. Furthermore, the study spans a fairly long period of time, during which surgical technique, implant design and adjuvant treatments have evolved to a certain degree and which is a cause for some inhomogeneity in our cohort. On the other hand, this allowed us to achieve a long follow-up period, and we have previously demonstrated that implant survivorship in our cohort did not differ for patients treated at different points during the study period [3]. Furthermore, we attempted to partially offset the impact of such an inhomogeneity by only including patients treated at a single institution and with a single modular megaprosthetic system.

We also acknowledge that we could only include a limited number of implants in some anatomic localizations, and some of our results might not be transferable to all sites of megaprosthetic reconstruction. Nonetheless, we chose to include all localizations as they represent the typical distribution of extremity bone sarcomas as seen in everyday practice. Finally, we investigated periprosthetic infections as a failure mode, the successful management of which depends on multiple factors such as microbiological findings, antibiotic therapy as well as host and soft tissue conditions, [22,23,24,25,26,27] which could not be evaluated in detail in the present analysis. However, we believe that this is balanced out by the large number of infectious megaprosthetic failures we were able to examine.

Our results suggest that the duration of the resection of a bone sarcoma and megaprosthetic reconstruction in a tertiary center is not itself an independent risk factor for the development of megaprosthetic infections. This contradicts the findings of two previous studies by Dhanoa et al. and Peel et al., which reported a significantly longer duration of primary surgery in patients with megaprostheses who went on to develop infections in two cohorts of 105 and 121 patients, respectively [13,14]. However, these studies on the one hand included patients with benign tumors and—in the study by Dhanoa et al.—non-oncological patients, the surgical treatment of which is generally both shorter and spares much more soft tissue compared to sarcoma patients [13]. Furthermore, both studies also included patients with pelvic tumors undergoing megaprosthetic reconstructions, which are associated with both a much longer duration of surgery and a much higher probability of postoperative infection compared to patients with extremity sarcomas [28]. The latter might also explain the somewhat high infection rate particularly in the study by Peel et al. of 28% [14]. Contrary to these studies, Cho et al. [15] investigated 62 patients undergoing proximal tibial replacement for malignant and locally aggressive bone and soft tissue tumors and did not find a correlation between a duration of surgery and infections, however this study again included patients with benign tumors as well as patients with bone metastases.

Our analysis also demonstrated that mechanical complications as a first implant failure were associated with a longer length of surgery. To our knowledge, such an association has not been described previously and given the retrospective study design, we can only speculate about potential causes. A longer duration of primary tumor surgery usually occurs in more extensive tumors that may require more time for dissection and may result in a more severe soft tissue damage. The resection of a greater amount of soft tissue may lead to a reduced implant support that might render the affected limb more prone to mechanical complications.

Another interesting finding of our study regarded the influence of surgical time on the probability of subsequent failure after the surgical treatment of the first prosthetic failure. One the one hand, we found no correlation between at the duration of primary surgery and the probability of subsequent failure, suggesting that the impact of the duration of primary surgery is mostly restricted to the first complication, and on the other hand, the duration of revision surgery for first infectious complication was significantly—and relevantly—shorter in patients with subsequent infections, compared to patients without subsequent infections. To our knowledge, no study has yet examined this aspect of revision surgery as a risk factor for further complications. In recent years aggressive debridement of bradytrophic tissue around the prosthesis during revision surgery has been proposed as a means to reduce the probability of subsequent megaprosthetic infections, and has also been shown to facilitate one-stage exchange procedures in patients with infected implants [5,16,24,29,30]. The longer duration of revision surgery in the group of patients without subsequent failures might, therefore, be considered to be a surrogate for the aggressiveness of the revision surgery, although we readily acknowledge the purely hypothetical nature of this suggestion in our cohort.

## 5. Conclusions

In conclusion, the duration of primary tumor surgery and megaprosthetic reconstruction at an experienced tertiary bone sarcoma center appears not to be associated with the risk of first megaprosthetic infection. On the other hand, a longer duration of first revision surgery for infection was associated with a lower risk for subsequent revisions. While this finding should be confirmed in an independent cohort and possible reasons should be evaluated in future studies, we believe that aiming for a shorter surgical duration in revision surgery at the expense of the meticulousness of the procedure might not be the optimal way to avoid further prosthesis revisions.

## Figures and Tables

**Table 1:** Patient demographics and surgical details for all patients excluding local tumor progression as a first failure mode, patients with a first failure excluding patients who underwent amputation for the first failure and patients with a second failure.

Variable	All Patients *n* = 568	Patients with a First Failure *n* = 230
Males	63% (*n* = 357)	69% (*n* = 159)
Diabetes	2% (*n* = 13)	2% (*n* = 5)
Smoking	8% (*n* = 46)	4% (*n* = 9)
Pathological fracture	10% (*n* = 55)	4% (*n* = 9)
Previous surgery	12% (*n* = 66)	14% (*n* = 31)
Cemented stem	24% (*n* = 136)	22% (*n* = 50)
Extra-articular resection	22% (*n* = 122)	27% (*n* = 61)
Reconstruction site		
Distal femur	38% (*n* = 218)	48% (*n* = 110)
Proximal tibia	17% (*n* = 96)	26% (*n* = 58)
Proximal femur	17% (*n* = 99)	8% (*n* = 19)
Proximal humerus	16% (*n* = 92)	7% (*n* = 16)
Total femur	4% (*n* = 21)	3% (*n* = 8)
Total knee	2% (*n* = 11)	3% (*n* = 8)
Total humerus	4% (*n* = 24)	3% (*n* = 6)
Distal humerus	1% (*n* = 7)	2% (*n* = 5)

**Table 2:** Oncological details for all patients excluding local tumor progression as a first failure mode, patients with a first failure excluding patients who underwent amputation for the first failure and patients with a second failure.

Tumor Entity	All Patients *n* = 568	Patients with a First Failure *n* = 230
High-grade osteosarcoma	52% (*n* = 295)	61% (*n* = 140)
Ewing sarcoma	15% (*n* = 87)	12% (*n* = 28)
Chondrosarcoma	13% (*n* = 74)	8% (*n* = 18)
Pleomorphic sarcoma	11% (*n* = 63)	8% (*n* = 19)
Low-grade osteosarcoma	3% (*n* = 16)	4% (*n* = 10)
Dedifferentiated chondrosarcoma	2% (*n* = 11)	2% (*n* = 5)
Others	4% (*n* = 22)	4% (*n* = 10)
Local radiation treatment	22% (*n* = 129)	20% (*n* = 46)
Preoperative	10% (*n* = 56)	6% (*n* = 14)
Postoperative	14% (*n* = 78)	15% (*n* = 34)
Systemic chemotherapy	79% (*n* = 450)	76% (*n* = 175)
Preoperative	73% (*n* = 415)	76% (*n* = 175)
Postoperative	78% (*n* = 444)	82% (*n* = 189)
Metastasized disease	30% (*n* = 170)	19% (*n* = 44)
Primary metastases	17% (*n* = 95)	13% (*n* = 29)
Died of disease	23% (*n* = 128)	14% (*n* = 31)

**Table 3:** Length of surgery for the different anatomic sites of reconstruction, displaying the differences between patients with or without implant failure and distinguishing between infectious and mechanical failures.

Variable	Rate of Failures	Median Length of the Initial Surgery in Minutes		
Anatomic Location and Types of Failure	% (*n*)	In Patients with Implant Failure	In Patients without Implant Failure	*p* (Mann–Whitney U-Test)
“Around the knee”	54% (176/325)	215	195	<0.0001
Mechanical	44% (142/325)	229	195	<0.0001
Infection	10% (34/325)	202	210	0.867
Distal femoral replacement	50% (110/218)	203	187	0.003
Mechanical	39% (85/218)	206	190	0.003
Infection	11% (25/218)	195	195	0.924
Proximal tibial replacement	60% (58/96)	240	220	0.127
Mechanical	53% (51/96)	235	220	0.538
Infection	7% (7/96)	270	227	0.097
Upper extremity	22% (27/123)	206	193	0.92
Mechanical	11% (13/123)	238	193	0.215
Infection	11% (14/123)	189	196	0.309
Lower extremity	45% (203/446)	225	210	0.001
Mechanical	36% (159/446)	235	209	<0.0001
Infection	10% (44/446)	203	215	0.716
Total bone or total knee	39% (22/56)	278	242	0.075
Mechanical	23% (13/56)	295	242	0.001
Infection	16% (9/56)	233	275	0.147

**Table 4:** Multivariate Cox regression analysis of risk factors for first prosthetic failure. * for the multivariate analysis the threshold value determined using Youden’s index was used as opposed to the metric value of the duration of surgery.

Variable	Hazard Ratio	*p*-Value	95% CI
Extra-articular resection	1.9	<0.001	1.4–2.6
Reconstruction length in millimeters	1	0.662	1–1
Duration of initial surgery (categorized) *	1.4	0.033	1.1–1.8
Diabetes	1.1	0.839	0.4–3
Postoperative radiation	1.3	0.164	0.9–2

**Table 5:** Multivariate Cox regression analysis of risk factors for second prosthetic failure. * for the multivariate analysis the threshold value determined using Youden’s index was used as opposed to the metric value of the duration of surgery.

Variable	Hazard Ratio	*p*-Value	95% CI
Extra-articular resection	1.5	0.110	0.9–2.4
Reconstruction length in millimeters	1	0.425	1–1
Diabetes	5.8	0.004	1.7–19
Duration of the initial surgery (categorized) *	0.9	0.521	0.6–1.4
Duration of the revision surgery (categorized) *	0.5	0.003	0.3–0.8
Postoperative radiation	2.5	0.001	1.5–4.4

## Data Availability

All relevant data is in the manuscript.

## Supplementary Materials

The following are available online at https://www.mdpi.com/article/10.3390/cancers13112510/s1, Table S1: Subtypes of structural failures for first and second prosthetic failures.
