# Supplementary material for: Does the Duration of Primary and First Revision Surgery Influence the Probability of First and Subsequent Implant Failures after Extremity Sarcoma Resection and Megaprosthetic Reconstruction?
Source: Cancers (Basel). 2021 May 21;13(11):2510. doi: 10.3390/cancers13112510 (PMC8196552; doi:10.3390/cancers13112510)
Supplement: Supplementary file 1 [file cancers-13-02510-s001.zip › cancers-1189761-after proof supplementary v2.pdf]

cancers MDPI

Supplementary Material: Does the Duration of Primary and First Revision Surgery Influence the Probability of First and Subsequent Implant Failures after Extremity Sarcoma Resection and Megaprosthetic Reconstruction?

Christoph Theil, Kristian Nikolaus Schneider, Georg Gosheger, Ralf Dieckmann, Niklas Deventer, Jendrik Hardes, Tom Schmidt-Braekling and Dimosthenis Andreou

Table S1. Subtypes of structural failures for first and second prosthetic failures.

**Structural Failure Type**	**% (*n*)**
First structural failures	37 % (84/230)
Failure of bushings/coupling	68% (57/84)
Implant breakage	14% (12/84)
Failure of supporting bone	18% (15/84)
Second structural failures	34%% (38/112)
Failure of bushings/coupling	92% (35/38)
Implant breakage	2% (1/38)
Failure of supporting bone	6% (2/38)
